# The Vaccination Papers Good Missionary Literature

**Published:** 1898-08

**Authors:** Charles E. Johnson

**Affiliations:** Sherman, Tex.


### THE VACCINATION PAPERS GOOD MISSIONARY LITERATURE.

SHERMAN, TEX., July 18th, 1898.

EDITOR OF THE HOMŒOPATHIC PHYSICIAN:

I consider Dr. Leverson's papers, as given us in THE HOMŒOPATHIC PHYSICIAN, of the utmost importance. And why any physician, and especially a homœopathic physician, should object to them is a mystery. My observation has led me to the firm conviction that vaccination is the direct cause of very many chronic ills. I take especial pains to have members of our School Board read your journal each month, and use each number as missionary literature, and as a result we have beaten the vaccinationists in our public schools. By all means give us as much light upon this subject as possible.

Fraternally,

CHAS. E. JOHNSON, M. D.

